# Molecular Survey and Spatial Distribution of *Rickettsia* spp. in Ticks Infesting Free-Ranging Wild Animals in Pakistan (2017–2021)

**DOI:** 10.3390/pathogens11020162

**Published:** 2022-01-26

**Authors:** Abid Ali, Shehla Shehla, Hafsa Zahid, Farman Ullah, Ismail Zeb, Haroon Ahmed, Itabajara da Silva Vaz, Tetsuya Tanaka

**Affiliations:** 1 Department of Zoology, Abdul Wali Khan University Mardan, Mardan 23200, Pakistan; shehla@awkum.edu.pk (S.S.); hafsa.zahid@awkum.edu.pk (H.Z.); farman.ullah@awkum.edu.pk (F.U.); ismailzeb@awkum.edu.pk (I.Z.); 2 Department of Biosciences, COMSATS University Islamabad (CUI), Islamabad 45550, Pakistan; Haroonahmed@comsats.edu.pk; 3 Centro de Biotecnologia, Universidade Federal do Rio Grande do Sul, Porto Alegre 91501-970, Brazil; itabajara.vaz@ufrgs.br; 4 Laboratory of Infectious Diseases, Joint Faculty of Veterinary Medicine, Kagoshima University, 1-21-24 Korimoto, Kagoshima 890-0065, Japan

**Keywords:** wild animals, *Rhipicephalus*, *Rickettsia*, Khyber Pakhtunkhwa, Pakistan

## Abstract

*Rickettsia* spp. associated with ticks infesting wild animals have been mostly neglected in several countries, including Pakistan. To address this knowledge gap, ticks were collected during 2017 to 2021 from wild animals including cats (*Felis chaus*), Indian hedgehogs (*Paraechinus micropus*), and wild boars (*Sus scrofa*). The collected ticks were morpho-molecularly identified and screened for the detection of *Rickettsia* spp. Morphologically identified ticks were categorized into four species of the genus *Rhipicephalus*: *Rhipicephalus haemaphysaloides*, *Rh. turanicus*, *Rh. sanguineus* sensu lato (s.l), and *Rh. microplus*. Among 53 wild animals examined, 31 were infested by 531 ticks, an overall prevalence of 58.4%. Adult female ticks were predominant (242 out of 513 ticks collected, corresponding to 46%) in comparison with males (172, 32%), nymphs (80, 15%) and larvae (37, 7%). The most prevalent tick species was *Rh. turanicus* (266, 50%), followed by *Rh. microplus* (123, 23%), *Rh. sanguineus* (106, 20%), and *Rh. haemaphysaloides* (36, 7%). Among the screened wild animals, wild boars were the most highly infested, with 268 ticks being collected from these animals (50.4%), followed by cats (145, 27.3%) and hedgehogs (118, 22.3%). Tick species *Rh. haemaphysaloides*, *Rh. turanicus*, and *Rh. sanguineus* were found on wild boars, *Rh. haemaphysaloides*, and *Rh. microplus* on cats, and *Rh. turanicus* on hedgehogs. In a phylogenetic analysis, mitochondrial cytochrome C oxidase 1 (*cox1*) sequences obtained from a subsample (120) of the collected ticks clustered with sequences from Bangladesh, China, India, Iran, Myanmar, and Pakistan, while *16S* ribosomal DNA (*16S rDNA*) sequences clustered with sequences reported from Afghanistan, Egypt, India, Pakistan, Romania, Serbia, and Taiwan. Among *Rickettsia* infected ticks (10/120, 8.3%), *Rh. turanicus* (7/10, 70%), and *Rh. haemaphysaloides* (3/10, 30%) were found infesting wild boars in the districts Mardan and Charsadda. The obtained rickettsial *gltA* gene sequences showed 99% and *ompA* gene sequences showed 100% identity with *Rickettsia massiliae*, and the phylogenetic tree shows *ompA* clustered with the same species reported from France, Greece, Spain, and USA. This study emphasizes the need for effective surveillance and control programs in the region to prevent health risks due to tick-borne pathogens, and that healthy infested wild animals may play a role in the spread of these parasites.

## 1. Introduction

Interactions between domestic and wild animals have increased due to urbanization, deforestation, and anthropogenic activities which enhance the risk of emergence of zoonotic diseases [1]. Ticks’ harmful effects are not only restricted to livestock and humans, but are also a major threat to wild animals and thus are important from a conservation point of view [2]. Moreover, wild animals serve as bridges for pathogen transmission between wildlife and humans. Tick-infested wild and domestic animals exchange ticks, and thus pathogenic organisms, upon sharing habitats [2]. 

Free roaming behavior of wild cats in search of food increases the chances of interaction with diverse habitats and animals, and also enhances the chances of exposure to different ticks and tick-associated pathogens. Cats have been found infested with different tick species including *Ixodes ricinus*, *I. hexagonus*, *I. trianguliceps* in Great Britain [3], *I. scapularis*, *I. pacificus*, *I. banksi*, *Amblyomma americanum*, *A. maculatum*, *Dermacentor occidentalis*, *Otobius megnini*, *I. affinis I. angustus*, *I. cookie*, *D. variabilis*, *Haemaphysalis longicornis* in USA [4,5,6], *Rhipicephalus sanguineus* in Pakistan [7] *A. testudinarium* in Japan [8], *D. albipictus*, *D. andersoni*, and *H. hystricis* in Belgium [9]. Tick-infested cats have been found infected with *Anaplasma* spp., *Borrelia* spp., *Babesia* spp., *Ehrlichia* spp., *Bartonella* spp., and hemoplasma species [3,10,11]. Hedgehogs occupy diverse habitats, mostly living in burrows, and studies of their potential as a host for ectoparasites are limited due to their nocturnal behavior [12]. Hedgehog domestication has become increasingly common in the last few decades, which has enhanced the risk of several tick-borne pathogens in humans and domesticated animals. Hedgehogs have been found infested with species of different tick genera including *Amblyomma* spp., *Dermacentor* spp., *Hyalomma* spp., *Haemaphysalis* spp., *Rhipicephalus* spp., and *Ornithodoros* spp. [13,14]. Hedgehogs infested by ticks may carry various tick-borne pathogens including tick-borne encephalitis virus, *Borrelia* spp., *Anaplasma marginale*, and *A. phagocytophilum* and may serve as a reservoir for various other unknown infectious agents [14,15,16]. The movement of wild boars towards suburban and urban areas has been observed, resulting in their interaction with domestic animals, spreading ticks and tick-associated pathogens [17]. Wild boars have been observed parasitized by different tick species such as *D. atrosignatus*, *D. steini*, *D. compactus*, *D. marginatus*, *D. reticulatus*, *Rh. turanicus*, *Rh. sanguineus*, *I. ricinus*, and *H. hystricis* in the Asian and Southeast Asian countries [18,19,20,21,22]. Reports have shown the occurrence of tick-borne pathogens such as *A. marginale*, *A. phagocytophilum*, and *B. burgdorferi* sensu lato (s.l.) associated with ticks infesting wild boar in Europe, Portugal, and Iran [14,23].

Surveillance of ticks in birds, reptiles, mammals, and vegetation has led to the identification of known and yet-to-be-described pathogens belonging to the genus *Rickettsia*. Several tick species including *Hya. anatolicum*, *Hya. hussaini* [24], *Rh. sulcatus*, *Rh. lunulatus*, *Rh. muhsamae*, *Rh. senegalensis*, *Rh. turanicus*, *Rh. sanguineus* [25,26], *D. reticulatus*, *H. punctata* [27], and *I. ricinus* [28] have been reported associated with *Rickettsia massiliae*. Infections caused by tick-borne *R. massiliae* in humans have been reported in various countries [28,29]. In Pakistan, research has been carried out on ticks collected from various mammals, reptiles, and birds [7,20,30,31,32,33]. Various tick-borne pathogens have been investigated in ticks infesting domestic animals. We have recently reported *R. massiliae* in *Rhipicephalus* ticks infesting equids [34]. However, studies so far have neglected to screen *Rickettsia* species associated with ticks infesting free-ranging wild animals in Pakistan. To fill this gap, the present study was designed to screen *Rickettsia* species in ticks infesting wild animals such as cats (*Felis chaus*), Indian hedgehogs (*Paraechinus micropus*), and wild boars (*Sus scrofa*) in selected districts of Khyber Pakhtunkhwa (KP), Pakistan.

## 2. Results

### 2.1. Ticks’ Morphological Description

**Figure 1:**
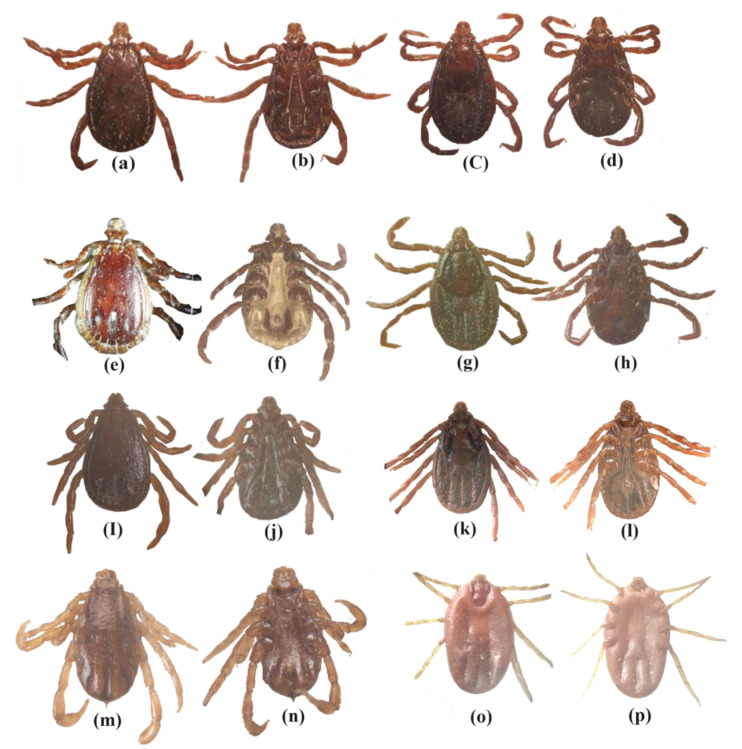
*Rhipicephalus haemaphysaliodes*: (**a**) male dorsal, (**b**) male ventral, (**c**) female dorsal, (**d**) female ventral; *Rhipicephalus sanguineus*: (**e**) male dorsal, (**f**) male ventral, (**g**) female dorsal, (**h**) female ventral; *Rhipicephalus turanicus*: (**i**) male dorsal, (**j**) male ventral, (**k**) female dorsal, (**l**) female ventral; *Rhipicephalus microplus*: (**m**) male dorsal, (**n**) male ventral, (**o**) female dorsal, (**p**) female ventral.

The collected ticks were identified according to their distinguishing features, e.g., basis capituli of male *Rh. haemaphysaloides* bear slightly sharp and pointed cornua and sickle-shaped adanal plates. The female *Rh. haemaphysaliodes* scutum has nearly the same length as width, with a slightly sinuous posterior margin. The genital opening is narrowly U-shaped on the ventral side. The width of capituli in male *Rh. sanguineus* sensu lato (s.l) is greater than its length with acutely-curved lateral angles of the basis capituli. Adanal plates are subtriangular or rounded posteriorly. The length of scutum in female *Rh. sanguineus* is more than its width, having a sinuous posterior margin. The posterior lip genital aperture is broad and U-shaped. Basis capituli of male *Rh. turanicus* have sharp and pointed cornua with comma-shaped cervical grooves. The adanal plates may be broad and shortened or sharp and longer posteriorly. The posterior margin of the scutum in female *Rh. turanicus* is distinctly sinuous, having a small genital aperture and U-shaped to broadly V-shaped posterior lip. Male *Rh. microplus* has a distinct cornua, and ventrally the coxa 1 bears long and distinct spurs. In female *Rh. microplus* the scutum is pear shaped, with a broader U-shaped genital aperture (Figure 1).

### 2.2. Tick Infestation and Wild Animals

Among 53 examined wild animals in Charsadda district (10 wild boars), Mardan (10 cats and 10 wild boars), Peshawar (11 cats and 4 hedgehogs), and Swabi (4 cats and 4 hedgehogs), 31 were infested with 531 ticks at different life stages (Table 1). Adult females were the most prevalent (number of ticks 242, corresponding to 46%) followed by males (172, 32%), nymphs (80, 15%) and larvae (37, 7%) (Table 1). The collected ticks were categorized into four species of the genus *Rhipicephalus*: *Rh. haemaphysaloides*, *Rh. microplus*, *Rh. sanguineus*, and *Rh. turanicus*. Overall prevalence of tick infestation among wild animals was 58.4%, with the highest tick burden being observed in wild animals of Mardan district (201, 37.7%) followed by Peshawar (153, 28.7%), Charsadda (112, 21.4%), and Swabi (65, 12.2%). A significant difference in tick infestation was observed among wild animals from different districts (*p* 0.0361). The most prevalent tick species was *Rh. turanicus* (266, 50%) followed by *Rh. microplus* (123, 23%), *Rh. sanguineus* (106, 20%), and *Rh. haemaphysaloides* (36, 7%). Wild boars were highly infested (268, 50.4%) by ticks including *Rh. turanicus* (148, 55%), *Rh. sanguineus* (106, 40%) and *Rh. haemaphysaloides* (14, 5%). Following wild boars, cats were infested by 145 ticks (27%) including *Rh. microplus* (123, 85%) and *Rh. haemaphysaloides* (22, 15%) (Table 1). Hedgehogs were the least infested, with 118 (22.2%) *Rh. turanicus* ticks. Districtwide tick infestation in wild animals was found to be statistically significant (*p* 0.0001).

### 2.3. Molecular Identification and Phylogeny of Ticks

The *cox1* sequence fragments obtained from morphologically identified *Rh. haemaphysaloides*, *Rh. sanguineus*, *Rh. turanicus* (wild boar), *Rh. turanicus* (hedgehog), and *Rh. microplus* were 521 bp, 603 bp, 778 bp, 659 bp and 780 bp, respectively. The *16S rDNA* sequence fragments were 388 bp and 390 bp for *Rh. haemaphysaloides* from cat and wild boar, respectively; 408 bp and 419 bp for *Rh. turanicus* from hedgehog and wild boar, respectively; and 340 bp for *Rh. sanguineus*, and 407 bp for *Rh. microplus*.

**Figure 2:**
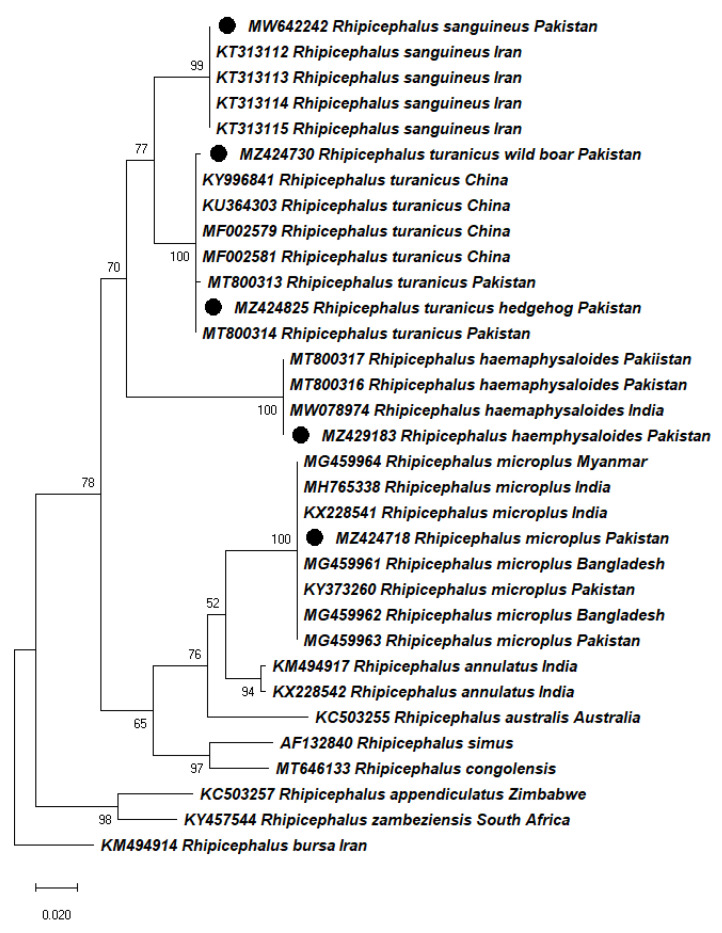
Phylogenetic tree (Maximum Likelihood) based on *cox1* gene partial sequences of the collected ticks *Rh. haemaphysaloides*, *Rh. turanicus*, *Rh. sanguineus*, and *Rh. microplus* in Khyber Pakhtunkhwa (KP) Pakistan. *Rhipicephalus bursa* was used as an outgroup. Bootstrap values are presented at each node (1000). GenBank accession numbers are followed by species name and country of collection at each terminal taxon. Sequences obtained in this study are labeled with black circles.

The BLAST analysis of partial *cox1* gene sequences from *Rh. haemaphysaloides*, *Rh. turanicus*, *Rh. sanguineus*, and *Rh. microplus* showed 98-100% identity with sequences of the same species previously reported from Bangladesh, China, Iran, India, Myanmar, and Pakistan. In a phylogenetic tree (Maximum Likelihood), the obtained sequences of *Rh. haemaphysaloides* clustered with previously reported same-species sequences from India (MW078974) and Pakistan (MT800316, MT800317); *Rh. turanicus* sequences clustered with reported same-species sequences from China (KY996841, KU364303, MF002579, MF002581), and Pakistan (MT800313, MT800314); *Rh. sanguineus* sequences clustered with reported same-species sequences from Iran (KT313112, KT313113, KT313114, KT313115); and *Rh. microplus* sequences clustered with reported same-species sequences from Bangladesh (MG459961, MG459962), India (MH765338, KX228541), Myanmar (MG459964), and Pakistan (KY373260, MG459963) (Figure 2). The sequence data were analyzed by different methods in MEGA-X and similar phylogenetic results were recovered (data not shown).

**Figure 3:**
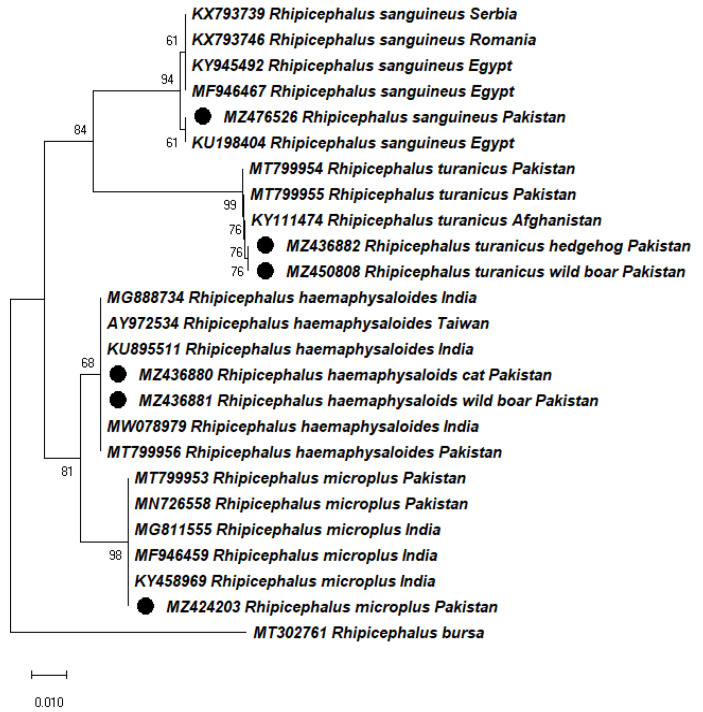
Phylogenetic (Maximum Likelihood) tree based on *16S rRNA* gene partial sequences of *Rh. haemaphysaloides*, *Rh. turanicus*, *Rh. sanguineus*, and *Rh. microplus* of Khyber Pakhtunkhwa (KP) Pakistan. *Rhipicephalus bursa* was used as an outgroup. Bootstrap values are presented at each node (1000). GenBank accession numbers are followed by species name and country of collection. Sequences obtained in the present study are labeled with black circles.

The BLAST analysis of *16S rDNA* sequences showed 97–100% identity with sequences previously reported for the respective tick species from Afghanistan, Egypt, India, Pakistan, Romania, Serbia, and Taiwan. In the phylogenetic tree, the obtained sequences of *Rh. haemaphysaloides* clustered with the same-species sequences from India (MG888734, KU895511, MW078979), Pakistan (MT799956), and Taiwan (AY972534), *Rh. turanicus* with Afghanistan (KY111474) and Pakistan (MT799954, MT799955), *Rh. sanguineus* with Egypt (KY945492, MF946467), Romania (KX793746), and Serbia (KX793739) and *Rh. microplus* with India (MG811555, MF946459, KY458969) and Pakistan (MT799953, MN726558) (Figure 3). The analysis of *cox1* and *16S rRNA* gene sequences and subsequent phylogenetic trees supported their monophyly, with identical sequences for each tick species found in previous reports from different countries.

The obtained *cox1* and *16S rRNA* gene sequences for each tick species were deposited in GenBank under accession numbers: MZ429183 and MZ436880, MZ436881 (*Rh. haemaphysaloides*), MZ424825, MZ424730, and MZ436882, MZ450808 (*Rh. turanicus*), MW642242 and MZ476526 (*Rh. sanguineus*), and MZ424718 and MZ424203 (*Rh. microplus*).

### 2.4. Detection of Rickettsia spp. in Ticks

Among the 120 ticks screened for *Rickettsia* spp., *Rh. turanicus* and *Rh. haemaphysaloides* collected from wild boars were found positive for rickettsial DNA, as determined by the amplification of both *gltA* (377 bp) and *ompA* (576 bp and 503 bp) partial sequences (Table 1). The overall prevalence of *Rickettsia* spp. was 8.3% (10/120) based on both *gltA* and *ompA* genes. The rickettsial DNA sequences were amplified from *Rh. turanicus* (7/10, 70%) and *Rh. haemaphysaloides* (3/10, 30%). The presence of *Rickettsia* spp. in the Charsadda district (6/10, 60%) was detected in *Rh. turanicus* (3/10, 30%) and *Rh. haemaphysaloides* (3/10, 30%), while in Mardan district (4/10, 40%) it was found in *Rh. turanicus* only. *Rickettsia* spp. was not detected in *Rh. microplus* and *Rh. sanguineus* ticks. 

**Figure 4:**
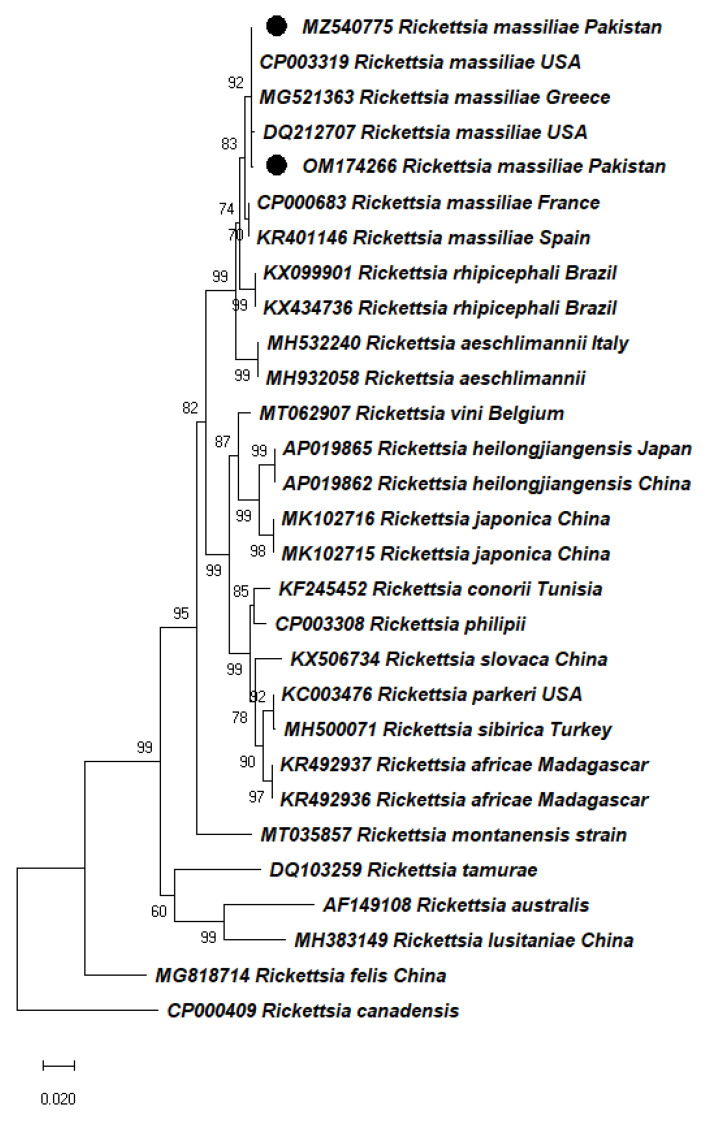
Maximum likelihood tree inferred from partial sequences of the *ompA* gene for *Rickettsia* spp. *Rickettsia canadensis* was used as an outgroup. Bootstrap values are presented at each node (1000). Accession numbers are followed by species and country name. Sequences obtained in the present study were labeled with black circles.

BLAST analysis of the obtained *gltA* gene (*Rickettsia*) sequences from *Rh. turanicus* and *Rh. haemaphysaloides* infesting wild boars showed 99% identity with *R. massiliae* sequences from China. On the other hand, *ompA* gene sequences detected in *Rh. turanicus* and *Rh. haemaphysaloides* infesting wild boars showed 99-100% identities with previously reported sequences of *R. massiliae* from France, Greece, Spain, and the USA. In a phylogenetic tree, the obtained sequences clustered with *R. massiliae* from France (CP000683), Greece (MG521363), Spain (KR401146), and USA (CP003319, DQ212707) (Figure 4). The resulting *gltA* and *ompA* sequences for *R. massiliae* were deposited in GenBank under accession numbers: (OM066912) and (MZ540775 and OM174266), respectively.

## 3. Discussion

Climate change, urbanization, and other anthropogenic activities have led to the destruction of wildlife habitats, which in turn has increased the chances of interaction between wild and domestic animals [19]. Diverse geographical regions comprising mountainous ranges and agro–wildlife localities serve as habitats for several wildlife species in Pakistan. Studies have been conducted on ticks infesting domestic animals, but research has often neglected ticks infesting wild animals in Pakistan. In this study, we inspected cats, hedgehogs, and wild boars for tick infestation in four districts of KP, Pakistan. The collected ticks were morpho-molecularly identified as *Rh. haemaphysaloides*, *Rh. turanicus*, *Rh. sanguineus* and *Rh. microplus* and screened for tick-associated *Rickettsia* species. *Rickettsia massiliae* was detected in *Rh. turanicus* and *Rh. haemaphysaloides* infesting wild boars in the Charsadda and Mardan districts.

Cats infested by ticks can bolster the dispersion of ticks and tick-borne pathogens to predisposed owners and other domestic animals [4]. In the current study, we observed the infestation of *Rh. microplus* and *Rh. haemaphysaloides* on cats. Cats and other wild animals generally acquire ticks from natural habitats and their inside access may create a risk of tick infestation to indoor domesticated animals, pets, and humans [4,6]. In this study, *Rh. turanicus* ticks were found infesting hedgehogs, and other tick species including *Rh. haemaphysaloides*, *Rh. sanguineus*, and *Rh. turanicus* were found infesting wild boars. *Rh. turanicus* has been previously reported as infesting hedgehogs in Iran [14] and Turkey [13]. Accordingly, *Rh. turanicus* infestation in hedgehogs, as observed in this study, provides evidence that hedgehogs are not accidental hosts for this tick. In wild boar, *Rh. sanguineus* and *Rh. turanicus* infestation has been previously reported in Sri Lanka [19], and *Rh. sanguineus* in KP, Pakistan [20]. The variety of ticks found infesting wild boar may be due to the free movement of this host and contact with other wild and domestic animals.

Morphological identification of the tick species was confirmed by sequencing fragments of mitochondrial genes (*cox1* and *16S rRNA*). Using morphology alone is insufficient for the precise identification of tick species due to morphological similarities, the presence of engorged as well as immature stages, and damaged specimens [20,35,36,37]. In several studies, both morphological and molecular identification of ticks have been implemented to achieve accurate taxonomic classification [34,36]. Molecular markers, including mitochondrial *cox1* and *16S rRNA*, have been reported in the successful determination of the evolution and phylogeny of ticks [37]. Among genetic markers, *16S rRNA* and *cox1* are useful for understanding interspecific phylogenetic and intraspecific genetic variabilities among ticks [20,37]. In this study, phylogenetic analysis of the identified *Rhipicephalus* species was performed using *cox1* and *16S rDNA* partial sequences, which revealed close evolutionary relationship with ticks of the same species reported from Afghanistan, Bangladesh, China, Egypt, India, Iran, Myanmar, Pakistan, Romania, and Taiwan.

Previously, *R. massiliae* has been detected in *Rhipicephalus* species including *Rh. haemaphysaloides*, *Rh. microplus*, *Rh. turanicus* [26,34], *Rh. sanguineus*, *Rh. sulcatus*, *Rh. lunulatus*, *Rh. muhsamae*, and *Rh. senegalensis* [25,27]. In this study *R. massiliae* was detected in *Rh. haemaphysaloides* and *Rh. turanicus* ticks collected from wild boars. *R. massiliae* has been described as infecting *Rh. turanicus* and *Rh. sanguineus* ticks collected from wild boars [26,38]. To date, there has been a lack of information about the detection of *R. massiliae* in *Rh. haemaphysaloides* ticks infesting wild boars. In Pakistan, the presence of *R. massiliae* was reported in *Rh. microplus*, *Rh. haemaphysaloides*, *Hya. anatolicum* and *Hya. hussaini* [24,34]. Free roaming of tick-infested wild boars into human residential areas can enhance the exposure of domestic animals and humans to rickettsial infection [17]. *Rh. turanicus* has been implicated as a vector of several medically important pathogens, such as *Babesia* spp., *Theileria* spp., *Anaplasma* spp., and *Rickettsia* spp. [14,39,40]. *Rh. haemaphysaloides* has been found infected with multiple pathogens comprising *Anaplasma* spp., *Babesia* spp., *Rickettsia* spp., *Borrelia* spp., and *Ehrlichia* spp. [34,41,42,43]. An increase in the free movement of tick-infested wild animals toward urban and suburban areas have resulted in the transmission of tick-associated *Rickettsia* spp. from wild animals to humans, pet animals, and livestock [19]. Therefore, the so-far neglected surveillance of tick-borne pathogens in ticks parasitizing wild animals demands immediate attention.

## 4. Materials and Methods

### 4.1. Ethical Approval

The experimental design of the present study was approved by the Advance Studies Research Board members of Abdul Wali Khan University, Pakistan (Dir/A&R/AWKUM/2018/1410).

### 4.2. Study Area

**Figure 5:**
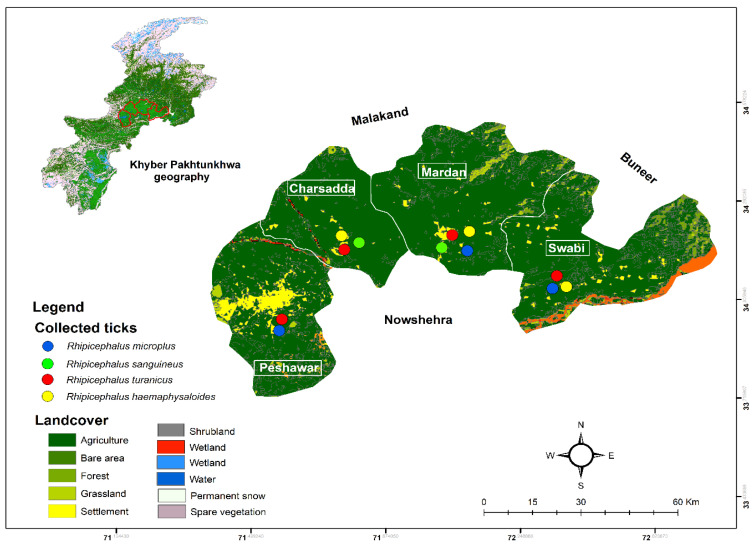
Vegetation map of the study area, indicating locations where different ticks were collected in each district of Khyber Pakhtunkhwa (KP), Pakistan.

The rural areas of the Charsadda, Mardan, Peshawar, and Swabi districts were selected for the collection of wild animals, including cats, Indian hedgehogs, and wild boars, during 2017–2021. The study area comprising selected districts in the KP northern province have their highest (33.4 °C) and lowest (11.7 °C) mean temperatures in July and December, respectively (climate-data.org) accessed on 27 May 2021. The exact geographical coordinates of sample locations were obtained using Global Positioning System (GPS) and added to the attribute table for tagging on the study area map using ArcGIS v. 10.3 (Figure 5).

### 4.3. Tick Collection and Morphological Identification

Wild animals including cats, hedgehogs, and wild boars found dead on highways, killed or captured by local farmers to secure their crops, were screened for ticks. Ticks found on the host body were carefully collected to avoid any damage to the specimens. All collected ticks were preserved in 100% ethanol. Morphological identification of the collected ticks was done using morphological features under Stereozoom microscope (BIOBASE, Jinan, China), by comparing with standard available morpho-taxonomic keys [44,45]. 

### 4.4. DNA Extraction and PCR

All ticks were morphologically identified, and 120 ticks comprising 10 specimens (different life stages) of each species from all districts were further processed for genomic DNA extraction (Table 1). Ticks were washed with distilled water followed by 70% ethanol and PBS for the removal of any surface contaminants. Washed ticks were individually kept in 1.5 ml tubes and dried in an incubator. Holes were made with needles, and the whole body of each tick was cut into small pieces using sterile scissors and homogenized by micro pestle for DNA extraction using phenol chloroform method [46]. The concentration of extracted DNA was measured using NanoQ (Optizen, Daejeon, South Korea), and samples were maintained at -20 ℃ for further analysis.

Mitochondrial cytochrome C oxidase 1 (*cox1*) and *16S* ribosomal RNA (*16S rRNA*) genes’ partial sequences were amplified for the molecular identification of ticks. The PCR was performed in a total volume of 25 µL reaction mixture comprised of 1 µL each forward and reverse primers (10 µM), 2 µL template DNA (50 ng), 8.5 µL PCR water, and 12.5 µL DreamTaq PCR Master Mix (2×) (Thermo Scientific, Waltham, MA, USA). Primers used in the present study are given in Table 2, and thermocycling conditions were set as previously described [47,48].

### 4.5. Detection of Rickettsia

All extracted genomic DNA samples were screened for the presence of any *Rickettsia* spp. targeting the amplification of rickettsial citrate synthase (*gltA*) and outer membrane protein (*ompA*) partial genes. The PCR reaction was performed in a total volume of 25 µL reaction mixture comprised of 1 µL each forward and reverse primers (10 µM), 2 µL template DNA (50 ng), 8.5 µL PCR water, and 12.5 µL DreamTaq PCR Master Mix (2×) (Thermo Scientific, Waltham, MA, USA). Primers used in the present study are given in (Table 2), and thermocycling conditions were set as previously described [49,50]. All genomic DNA samples that yielded visible amplicons for *gltA* PCR were subjected to second PCR assay for the amplification of *ompA* gene. The amplified PCR products were electrophoresed on 1.5% agarose gel and results were visualized under UV light using a GelDoc (UVP BioDoc-It imaging system, Upland, CA, USA).

### 4.6. DNA Purification and Sequencing

Prior to sequencing, the positive PCR products were purified with GeneClean II DNA purification Kit (Qbiogene, Illkirch, France) following the protocol provided by the manufacturer. All 120 purified PCR products for each *cox1* and *16S rRNA* gene of ticks, and 10 positive samples for each *gltA* and *ompA* of *Rickettsia* spp. were sent for bidirectional sequencing (Macrogen Inc., Seoul, South Korea).

### 4.7. Phylogenetic Analysis

The obtained sequences were trimmed in SeqMan V. 5.00 (DNASTAR) for the removal of unnecessary nucleotides and primer contamination. Redundant sequences (100% identity) were excluded from further analysis. Sequences with maximum identities were retrieved from NCBI (National Center for Biotechnology Information) using BLAST (Basic Local Alignment Search Tool) [51]. The obtained sequences were aligned in BioEdit V. 7.0.5 [52]. Phylogenetic trees were constructed in MEGA X [53], and different phylogenetic methods (Maximum likelihood, Neighbor-Joining, Minimum-Evolution, Parsimony, and UPGMA) were tested for consistency, efficiency, and robustness. The Maximum likelihood method was used for the phylogenetic tree, with bootstrap 1000 replicates, and an outgroup was used for estimating tree stability and validity, respectively. Finally, the sequences of *cox1*, *16S rDNA*, *gltA* and *ompA* were submitted to NCBI.

### 4.8. Statistical Analysis

The recorded data was organized in spreadsheets using Microsoft Excel V. 2016 (Microsoft). A chi-square test was performed using GraphPad prism software V. 5.00 (GraphPad Software Inc) considering a significant *p* value < 0.05.

## 5. Conclusions

The present study reported tick infestation in wild animals in KP, Pakistan, and for the first-time detected *R. massiliae* in *Rh. turanicus* and *Rh. haemaphysaloides* ticks infesting wild boars in Charsadda and Mardan. These results improve our knowledge of the circulation of *R. massiliae* in *Rhipicephalus* ticks infesting both domestic and wild animals. These findings reinforce the need to further understand the diversity of ticks infesting wild animals, tick-associated *Rickettsia* spp. and other pathogens across the country.

## Figures and Tables

**Table 1:** Abundance of ticks, and screening for rickettsial DNA associated with ticks infesting wild animals.

Districts	Host	Tick Species	Examined Hosts (%)	Infested Hosts (%)	Collected Ticks (%)	Tick Life Stages	Ticks Molecularly Analyzed *	*Rickettsia gltA* and *ompA*
Mardan	Cats	*Rh. microplus* *Rh. haemaphysaloides*	10 (18.8)	4 (40)	33 (73.3) 12 (36.6)	14F, 11M, 5N, 3L	8F, 2N	0
						5F, 4M, 3N	5F, 2M, 3N	0
	Wild boar	*Rh. turanicus* *Rh. sanguineus*	10 (18.8)	9 (90)	90 (57.6) 66 (42.4)	32F, 28M, 21N, 9L	6F, 4N	4
						27F, 19M, 13N, 7L	7F, 3N	
Peshawar	Cats	*Rh. microplus*	11 (20.7)	6 (54.5)	55 (36)	18F, 15M, 12N, 10L	8F, 2N	0
	Hedgehogs	*Rh. turanicus*	4 (7.54)	2 (50)	98 (64)	64F, 34M	10F	0
Charsadda	Wild boar	*Rh. turanicus* *Rh. sanguineus* *Rh. haemaphysaloides*	10 (18.8)	5 (50)	58 (52) 40 (36) 14 (12)	21F, 17M, 14N, 6L 19F, 17M, 4N	9F, 1N 8F, 2N	3 0
						6F, 4M, 2N, 2L	6F, 2M, 2N	3
Swabi	Cats	*Rh. microplus*	4 (7.54)	3 (75)	35 (54)	19F, 12M, 4N	8F, 2N	0
		*Rh. haemaphysaloides*			10 (15.3)	5F, 3M, 2N	5F,3M,2N	0
	Hedgehogs	*Rh. turanicus*	4 (7.54)	2 (50)	20 (30.7)	12F, 8M	6F, 4M	0
Total			53 (100)	31 (58.4)	531 (Mean 44.25)	242F, 172M, 80N, 37L	86F, 11M, 23N Total: 120	10(8.3%)

Note: F = Adult females, M = males, N = nymphs, L = larvae, * ticks molecularly tested and screened for *Rickettsia* spp.

**Table 2:** Primers used for the amplification of ticks and rickettsial DNA.

Organism	Gene	Primer	Sequence	Amplicon bp	References
Tick	*cox 1*	cox1F	GGAACAATATATTTAATTTTTGG	850	[47]
		cox1R	ATCTATCCCTACTGTAAATATATG		
	*16S*	16S+1	CCGGTCTGAACTCAGATCAAGT	460	[48]
		16S-1	GCTCAATGATTTTTTAAATTGCTGT		
*Rickettsia* spp.	*gltA*	CS-78	GCAAGTATCGGTGAGGATGTAAT	401	[49]
		CS-323	GCTTCCTTAAAATTCAATAAATCAGGAT		
	*ompA*	Rrl9O.70	ATGGCGAATATTTCTCCAAAA	631	[50]
		Rr190.701n	GTTCCGTTAATGGCAGCATCT		

## Data Availability

Details regarding data supporting reported results can be found https://www.ncbi.nlm.nih.gov/nuccore/?term= (accessed on 26 December 2021).
